# Molecular basis for the interaction between human choline kinase alpha and the SH3 domain of the c-Src tyrosine kinase

**DOI:** 10.1038/s41598-019-53447-0

**Published:** 2019-11-19

**Authors:** Stefanie L. Kall, Kindra Whitlatch, Thomas E. Smithgall, Arnon Lavie

**Affiliations:** 1 Department of Biochemistry and Molecular Genetics, University of Illinois at Chicago, Chicago, Illinois 60607 USA; 2 Department of Microbiology and Molecular Genetics, University of Pittsburgh School of Medicine, Pittsburgh, Pennsylvania 15219 USA; 3 The Jesse Brown VA Medical Center, Chicago, Illinois 60612 USA

**Keywords:** Proteins, Structural biology, Structural biology

## Abstract

Choline kinase alpha is a 457-residue protein that catalyzes the reaction between ATP and choline to yield ADP and phosphocholine. This metabolic action has been well studied because of choline kinase’s link to cancer malignancy and poor patient prognosis. As the myriad of x-ray crystal structures available for this enzyme show, chemotherapeutic drug design has centered on stopping the catalytic activity of choline kinase and reducing the downstream metabolites it produces. Furthermore, these crystal structures only reveal the catalytic domain of the protein, residues 80–457. However, recent studies provide evidence for a non-catalytic protein-binding role for choline kinase alpha. Here, we show that choline kinase alpha interacts with the SH3 domain of c-Src. Co-precipitation assays, surface plasmon resonance, and crystallographic analysis of a 1.5 Å structure demonstrate that this interaction is specific and is mediated by the poly-proline region found N-terminal to the catalytic domain of choline kinase. Taken together, these data offer strong evidence that choline kinase alpha has a heretofore underappreciated role in protein-protein interactions, which offers an exciting new way to approach drug development against this cancer-enhancing protein.

## Introduction

Choline kinase is the enzyme responsible for the conversion of choline (Cho) to phosphocholine (pCho) via the transfer of the gamma-phosphoryl group of ATP^1^. It is the first enzyme in the Kennedy Pathway, ultimately leading to the production of phosphatidylcholine, a key component of biological membranes. Choline kinase exists as two isoforms, alpha (ChoKα 1/2) and beta^2^. The alpha and beta isoforms are structurally similar and have sequence identity of 59%. However, the alpha isoform has a longer N-terminal sequence that shares no identity with the beta isoform; it also has a splice variant where residues 155–172 are missing (denoted ChoKα2; here we discuss the unspliced variant, ChoKα1, hereafter called ChoKα). ChoKα has 457 residues. However, crystal structures of the alpha isoform reveal only residues 80–457 (designated here as the catalytic domain), which suggests disorder in the first 79 residues of the enzyme^3^.

**Figure 1:**
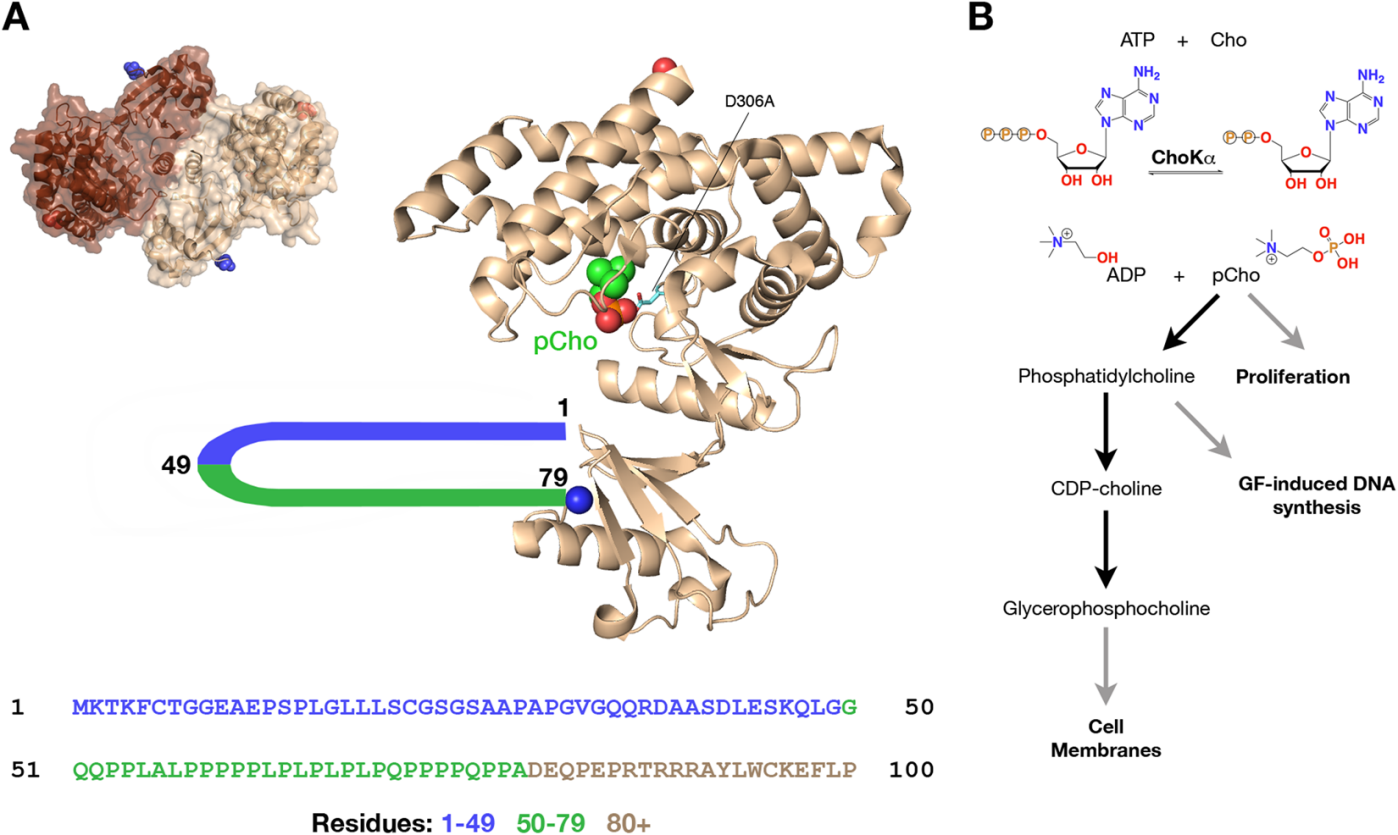
Crystal structure, N-terminal amino acid sequence and reaction mechanism of choline kinase alpha. (**A**) Crystal structure of ChoKα functional dimer (PDB ID 2CKQ) only shows order for residues 80–457 (*left*). The ribbon diagram of a single monomer is shown on the right.The blue sphere represents the first visible residue at the N-terminus while the red sphere represents the C-terminal residue, Val457. Asp306 (cyan, indicated with black line) is responsible for the transfer of the gamma phosphoryl group to choline to yield pCho (bright green). Residues 1–49 (blue) and 50–79 (green) do not appear in crystal structures; residues 50–79 contain a proline-rich region. (**B**) ChoKα catalyzes the conversion of choline (Cho) to pCho using ATP as a phospho-donor. Increased cellular pCho levels lead to increased cellular proliferation; other downstream products of the Kennedy pathway include phosphatidylcholine, which stimulates growth factor-induced DNA synthesis; and glycerophosphocholine, which is incorporated into cell membranes and is necessary for cell proliferation.

Structural studies and kinetic analysis have revealed important details about the mechanism of ChoKα. The enzyme active site consists of a deep pocket, with distinct sites for choline and ATP binding. Different enzyme conformations are associated with pCho and ATP binding^3^. The catalytic residue responsible for the transfer of the gamma-phosphoryl group of ATP is Asp306 (Fig. 1A)^4^. Mutation of this critical residue to alanine completely ablates enzymatic activity^5^. Studies of the homologous *Plasmodium falciparum* ChoK indicate that the enzyme undergoes a ping-pong mechanism, where ATP is first converted to ADP, yielding a covalent phosphoenzyme intermediate, followed by conversion of choline to pCho^6^.

ChoKα is upregulated in many tumor types, including breast, lung, colorectal, and prostate cancers^7^. The higher expression of choline kinase in cancer cells has been linked to poorer prognosis and outcomes in patients^8,9^. Likewise, the resultant increase in pCho bioavailability has been linked to higher tumor grade and a more aggressive phenotype^10^. Furthermore, upregulation of ChoKα leads to an increase in other downstream products of the Kennedy pathway, which are also linked to cancer progression (Fig. 1B)^11^. Due to its strong correlation with cancer and poorer patient prognosis, ChoKα has been a prime target for drug development studies. Many structures exist demonstrating how inhibitors bind to ChoKα, and several generations of drug candidates have been developed to inhibit this clinically relevant enzyme^12–14^.

Despite the successful development of potent and selective ChoKα inhibitors, cellular studies show that inhibition of the enzyme does not necessarily lead to apoptosis of cancer cells, but instead to cell senescence^15^. Furthermore, once the inhibitor is removed from the cells, they revert to their original phenotype^16^. Yet, ablation of ChoKα using siRNA in those same cell types leads to apoptosis^17^. Furthermore, while RNAi methods inhibit many of the negative downstream signaling events associated with ChoKα-driven cancer progression, downstream metabolites of the Kennedy pathway can selectively restore some of these signaling pathways^18^. Thus, there is a disconnect between the efficacy of kinase inhibitors *in vitro* and their usefulness as chemotherapeutic agents in cell-based models.

The key difference in the cellular responses to kinase inhibition vs. protein knockdown studies may lie in an additional, non-catalytic role of ChoKα^4^. Strong evidence for this hypothesis comes from studies of the epidermal growth factor receptor (EGFR) and c-Src tyrosine kinases, which are well known to cooperate in many types of cancer^10,19–21^. Miyake & Parsons demonstrated that ChoKα associates with EGFR in a manner dependent upon c-Src in a wide range of breast cancer cell lines and immortalized mammary epithelial cells^5^. ChoKα was phosphorylated by c-Src on Tyr197 and Tyr333, and over-expression of EGFR and c-Src increased the enzymatic activity and protein levels of ChoKα. This c-Src-dependent association between ChoKα and EGFR strongly suggests that ChoKα may serve both scaffolding and signaling functions in the context of this oncogenic pathway. However, the molecular basis for c-Src interaction with ChoKα is not known.

c-Src is the prototype of a large family of non-receptor tyrosine kinases^22^, and consists of a myristoylated unique region (residues 1–50), regulatory SH3 (84–145) and SH2 domains (151–248), and a kinase domain (270–523) with a negative regulatory tail^23^. Phosphorylation of Tyr527 in the negative regulatory tail induces intramolecular engagement of the SH2 domain, which is essential for suppression of kinase activity. The inactive conformation is stabilized by a second protein-protein interaction involving the SH3 domain, which binds to a polyproline type II helix formed by the SH2-kinase linker^24^. Src activation results from *trans*-interactions with substrates and other proteins through its SH3 and SH2 domains, which perturbs their negative regulatory influence on the kinase domain. In this way, protein-protein interaction and kinase activation are intimately linked. Active c-Src has many downstream targets involved in processes related to oncogenesis, including angiogenesis^25^, proliferation^26^, survival^27^, and cell motility^28^; these signaling pathways are often mediated by protein-protein interactions involving SH3 and SH2 domains^29^.

Though residues 1–79 of ChoKα have not been resolved in existing crystal structures, the region defined by amino acids 51–75 is remarkably proline-rich (Fig. 1A). Given the previous observation that c-Src is essential for the interaction of the EGFR with ChoKα^5^, we speculated that this proline-rich region of ChoKα may represent a docking site for c-Src via its SH3 domain. Here, we used complementary biophysical and biochemical methods to delineate the specific manner in which c-Src and ChoKα interact with each other. Co-precipitation assays and surface plasmon resonance studies offer strong evidence that ChoKα interacts with c-Src through its SH3 domain, and that other domains of Src may enhance this interaction. We also solved the X-ray crystal structure of a c-Src SH3 domain-ChoKα fusion peptide encompassing residues 60–69 of the ChoKα proline-rich region. This structure provides clear evidence that the ChoKα proline-rich region mediates interaction with the c-Src SH3 domain and provides the first structural view of this unique ChoKα protein binding motif.

## Results

### Biophysical studies reveal the importance of ChoKα residues 49–79 for the interaction with the SH3 Domain of c-Src

X-ray crystal structures have provided important insight into the substrate binding sites present in ChoKα. Though such studies have greatly advanced the mechanistic understanding of enzyme catalysis, they also show that only residues 80–457 are ordered in the protein (Fig. 1A). In our investigation of the protein-binding role of ChoKα, we considered those residues that do not appear in the existing ChoKα crystal structures (Fig. 1A). First, we compared the c-Src binding potential of ChoK as the full-length (FL) enzyme or with the first 49 (∆49) or 79 (∆79) residues truncated. Note that the FL and Δ49 variants include the poly-proline region, whereas the Δ79 variant does not. All constructs extended up to the C-terminal residue, Val457.

**Figure 2:**
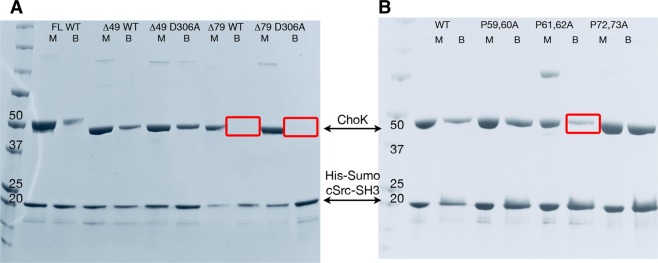
The SH3 domain of c-Src interacts with residues 50–79 in ChoKα, and requires prolines 61 and 62. (**A**) 1:1 mixture of purified ChoKα truncation constructs and of His-SUMO-c-Src (84–137) at 25 µM in 100 µl was washed three times with size exclusion purification buffer containing 30 mM imidazole. Both the active (WT) and kinase-dead mutants (D306A) were analyzed, demonstrating the enzyme activity has no impact on the protein binding behavior. M = protein mixture, B = bead-bound pull-down. Red boxes indicate where ChoKα would be expected. (**B**) Analysis of the proline-rich region between residues 50 and 79 uncovered several putative PxxP motifs that could be responsible for this interaction. Dual proline mutants were made in the background of ChoKαΔ49 to interrupt the predicted polyproline helix and experiments with co-precipitation experiments were repeated as described, showing a ~70% decrease in binding when prolines 61 & 62 were mutated to alanine (red box). Molecular weights for the constructs as follows: ChoKαFL = 52.2 kDa; ChoKαΔ49 = 47.7 kDa; ChoKαΔ79 = 44.4 kDa; His-SUMO-c-Src (84–137) = 19.6 kDa.

The full length and truncated versions of ChoKα were expressed and purified, along with the His-SUMO-tagged SH3 domain of c-Src. These purified proteins were combined and then assayed for co-precipitation using nickel-charged beads, followed by subsequent SDS-PAGE analysis. Initial studies revealed that while the FL and Δ49 constructs reliably and repeatedly co-precipitated with His-SUMO-c-Src-SH3, the Δ79 construct did not (Fig. 2A). Interestingly, the interaction between c-Src and ChoKα appeared to be stronger for the Δ49 deletion construct compared to the FL construct. In other words, the region between the N-terminus and residue 49 seems to weaken the interaction with the c-Src protein. This suggests that additional components may be required for achieving the full interaction capacity between c-Src and ChoKα. These could be proteins such as EGFR, as suggested by Miyake and Parsons^5^, or transient structural modifications due to intermediary ChoKα phosphorylated forms, as described by Zimmerman *et al*.^6^.

Notably, the c-Src - ChoKα interaction occurred with the enzymatically dead D306A variant of ChoKαΔ49 (Fig. 2A), indicating that the association is independent of ChoKα activity. Control experiments using FL ChoKαΔ verified that this interaction was not due to the His-SUMO tag, and was specific to the c-Src-SH3 domain vs. the SH3 domain of MLK3, selected as a first-pass negative control due to its involvement in unrelated pathways^30^ (Fig. S1A).

Because SH3 domains have been shown to interact with specific PxxP motifs, we selected a few proline-rich regions of ChoKα between residues 50 and 75 for further investigation. Double alanine mutants were made in order to disrupt the polyproline helix formed by this region^31^. In order to determine the specific region responsible for this interaction, three pairs of proline to alanine mutants were introduced in the background of the Δ49 ChoKα protein: P(59,60)A, P(61,62)A, and P(72,73)A. Co-precipitation experiments showed that the P(59,60)A and P(72,73)A proteins retained interaction with the c-Src SH3 domain, while binding to the P(61,62)A mutant was substantially reduced (Fig. 2B).

Specifically, we integrated the intensity of the bands on the gel and calculated the ratio between the ChoKα band intensity (i.e. the protein being pulled down) and that of the His-SUMO-cSrcSH3(87–137) band (i.e. the protein bound to the beads); the obtained ratios were 0.69, 0.70, 0.20, and 0.88 for WT, P(59,60)A, P(61,62)A, and P(72,73)A, respectively. Hence, only for the P(61,62)A mutant do we see a lower ratio, which represents a ~70% decrease in binding for the P(61,62)A mutant compared to the other P → A mutants.

Though some residual binding is seen for the P(61,62)A construct, it is clear that the overall affinity of c-Src-SH3 for this mutant is reduced. Additionally, pull-downs using the Δ62ChoKα construct exhibited no interaction with c-Src (Fig. S1B), which may imply that the retention of nearby prolines (such as P59 and P60) in the double alanine mutant is enough to allow some protein-protein interaction to occur. Interestingly, both the P(61,62)A and the Δ62 were difficult to purify and less soluble than their FL, Δ49, or Δ79 counterparts, as well as the P(59,60)A or P(72,73)A constructs.

**Figure 3:**
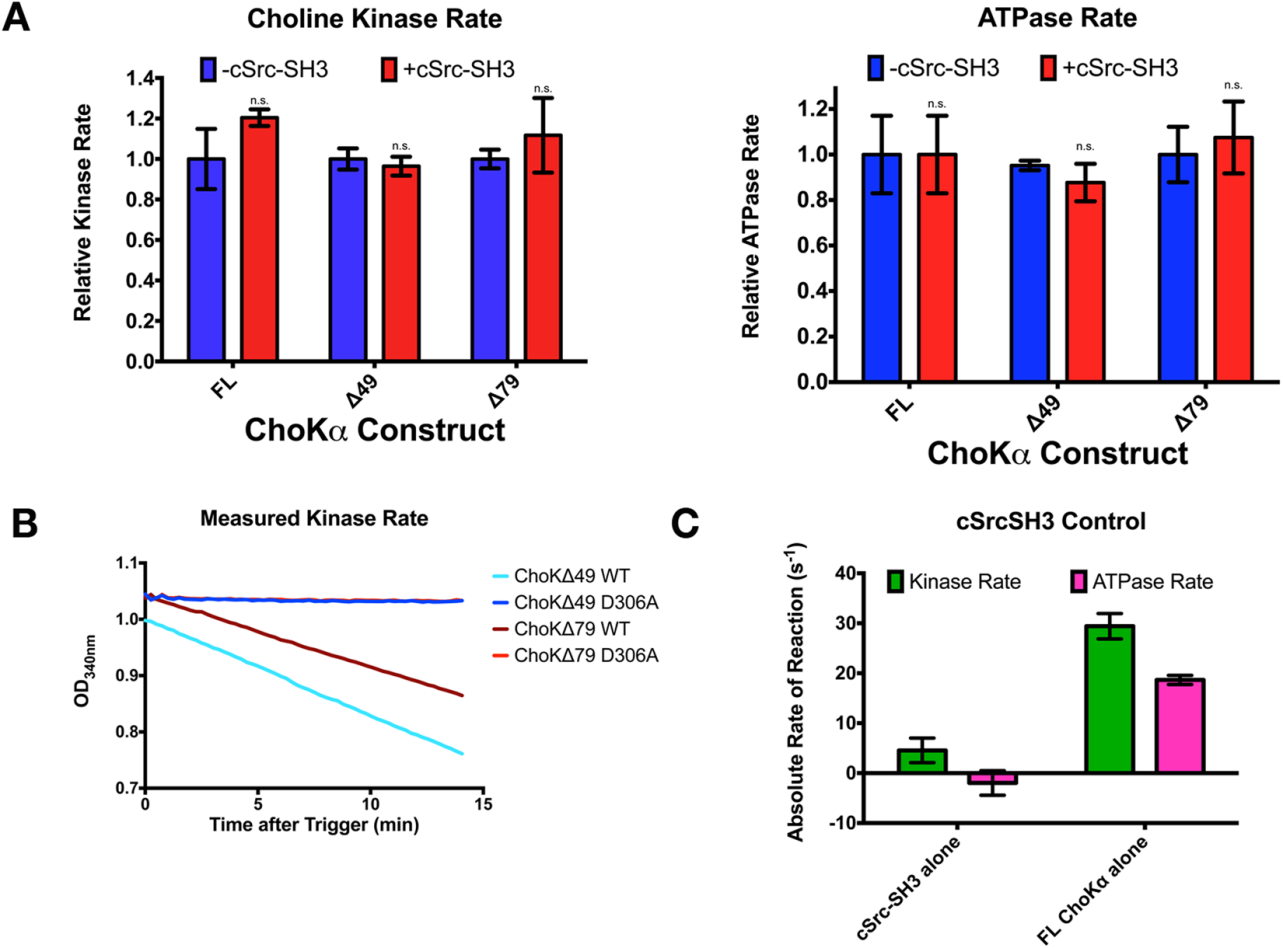
Interaction with the c-Src SH3 domain does not affect ChoKα activity. (**A**) The Choline Kinase rate (left) and the ATPase rate (right) of the constructs are unaffected by the presence of the SH3 domain of c-Src. (**B**) Kinase dead mutants show no activity compared to similar activity rates for the Δ49 and Δ79 wild-type enzymes. (**C**) The SH3 domain of c-Src has effectively no kinase or ATPase activity measured via this assay compared to full-length wild type choline kinase.

We next wanted to ascertain whether the interaction between ChoKα and the c-Src SH3 domain impacts the choline kinase enzymatic activity. Kinetic analysis was performed for each WT, active construct in the presence or absence of c-Src-SH3, and no significant differences in activity were revealed, for either the rate of choline phosphorylation or the rate of ATP hydrolysis. ChoKα has significant ATPase activity (Fig. 3A). The choline kinase rate of both the Δ49 and Δ79 constructs was 21.3 ± 3.2 and 19.5 ± 1.0 s^−1^, respectively, while as expected there is no activity for the D306 mutants (Fig. 3B). There was no discernable impact on activity in the presence of c-Src-SH3 alone compared to the wild type FL ChoKα enzyme (Fig. 3C). Thus, we concluded that this protein-protein interaction has no influence on enzymatic activity per se.

Taken together with the pull-downs of the D306A kinase-dead mutants, this was evidence that the enzymatic and protein binding functions of ChoKα are unrelated to each other, though it does not preclude the possibility that further interactions modulate ChoKα activity *in vivo*. For example, interaction with full-length Src may affect ChoKα activity, as may phosphorylation of ChoK by Src on Tyr333, as reported by Parsons^5^.

### Surface plasmon resonance (SPR) analysis suggests selective interaction between ChoKα and the SH3 of c-Src

**Figure 4:**
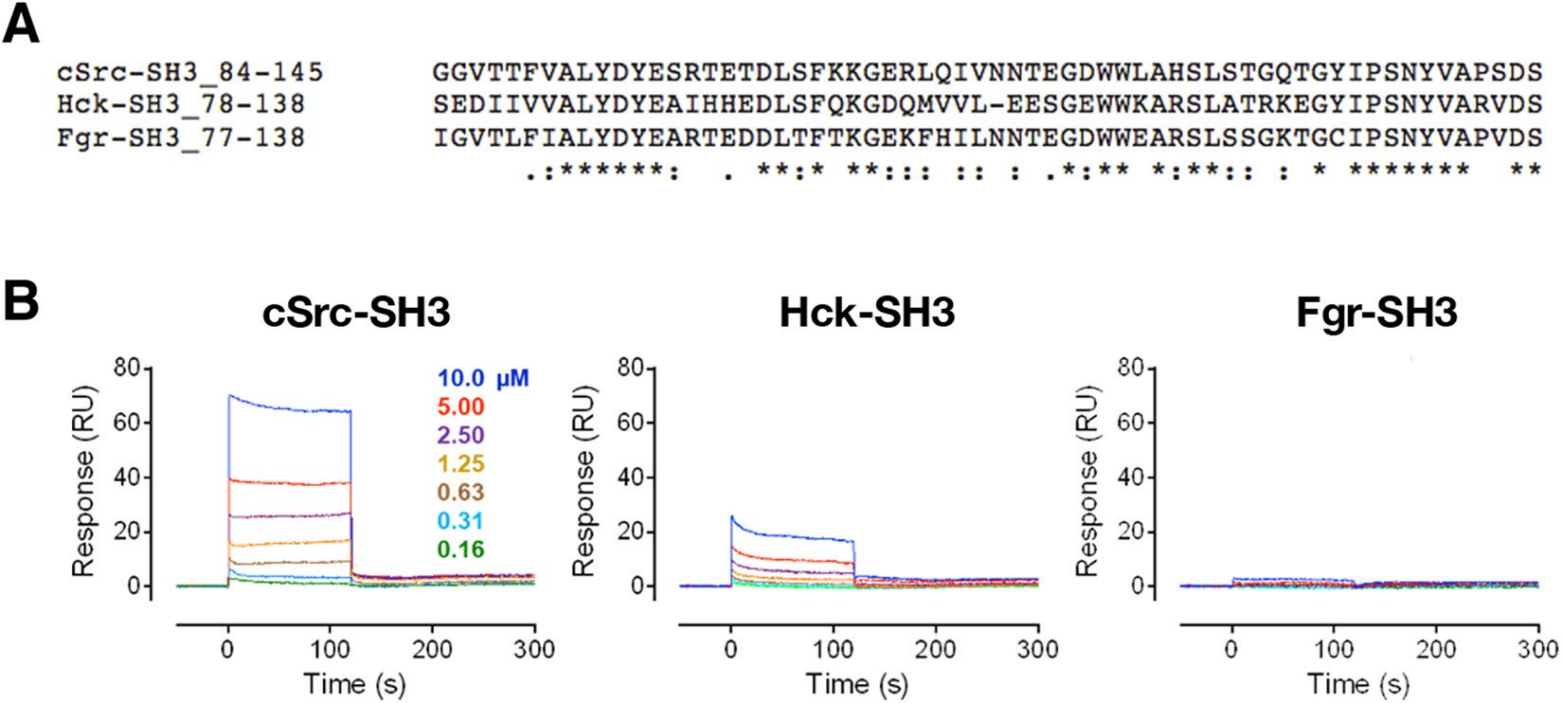
Selective interaction of ChoKα with the c-Src SH3 domain. (**A**) Alignment of the c-Src, Hck and Fgr amino acid sequences. Hck-SH3 has 51% sequence identity to c-Src-SH3; while the Fgr-SH3 has 73% identity. All three SH3 domains are known to interact with PxxP motifs and to have similar secondary structures. (**B**) SPR analysis of wild-type ChoKα interaction with the c-Src, Hck and Fgr SH3 domains. Recombinant SH3 domains were immobilized on a Biacore CM5 carboxymethyl dextran biosensor chip, and purified ChoKα was injected in triplicate over the range of concentrations shown (one trace shown at each concentration for clarity). Once equilibrium was reached, interactions were followed by a 2 min dissociation phase. A plot of the extent of interaction at each SH3 domain concentration was then fitted directly to determine the steady-state K_D_ value for Src and Hck.

SH3 domains exist in a wide variety of kinases, adaptors and other signaling proteins, and share a similar overall structure despite some amino acid sequence diversity. Since most SH3 domains bind proline-rich motifs that adopt polyproline type-II helices, we investigated whether the ChoKα/c-Src-SH3 interaction was specific, or whether SH3 domains from other Src-family members might interact as well. For these experiments, the recombinant SH3 domains from c-Src as well as the Src-family members Hck and Fgr (Fig. 4A) were immobilized on each channel of a single SPR chip, followed by injection of full-length wild-type ChoKα. The strongest responses were observed with the c-Src SH3 domain and yielded a steady-state K_D_ value of 14.2 µM (Fig. 4B). Interaction with the Hck SH3 domain was also observed, but the extent of binding, as well as the K_D_ value, were both lower (K_D_ = 25.9 µM). No interaction was observed with the Fgr SH3 domain over the same range of ChoKα concentrations. These findings support some degree of ChoKα selectivity for the c-Src SH3 domain, even among closely related SH3 domains within the c-Src family.

### The interaction between ChoKa and the c-Src-SH3 requires an intact polyP sequence preceding PL repeats

**Figure 5:**
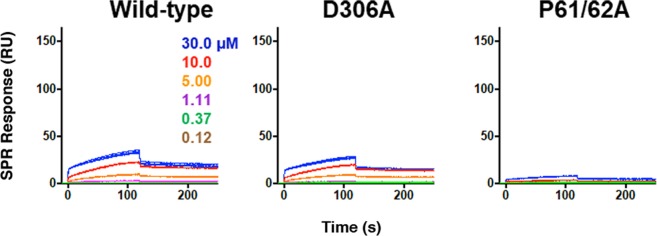
ChoKα Δ49 binding to c-Src-SH3 requires an intact polyproline sequence preceding PL repeats motif but not ChoK kinase activity. The recombinant wild-type and mutant ChoKα Δ49 proteins indicated at the top were immobilized on a Biacore CM5 carboxymethyl dextran chip, and the Src SH3 protein was injected in triplicate over the range of concentrations shown. Interaction was recorded for 2 min, followed by a 2 min dissociation phase. The resulting sensorgrams were best-fit by a two-state induced-fit model, and the resulting K_D_ values against ChoKα Δ49-WT and Δ49–306A are 2.4 × 10^−6^ M and 1.8 × 10^−6^ M, respectively. Insufficient interaction was observed with the Δ49-P61/62 A mutant to allow curve fitting.

We next explored whether the data obtained from previous pull-down experiments could be validated with additional biophysical analysis. For these studies, the wild-type, D306A, and P(61,62)A forms of ChoKα (each in the background of the Δ49 construct) were immobilized on a single SPR chip. The Src SH3 domain was tested as an analyte against the different ChoKα constructs. Under these SPR conditions, the Src SH3 domain interacted with both the wild-type and kinase-dead mutants of ChoKα Δ49 (Fig. 5). For both interactions, the K_D_ values were in the 2 µM range, which is consistent with published reports^32^ for optimized peptide ligands for the Src SH3 domain. In contrast, the P(61,62)A ChoKα Δ49 mutant showed very little interaction with the Src SH3 domain, consistent with the pull-down data shown in Fig. 2. The difference in binding kinetics and K_D_ values between this and the reverse interaction via SPR may reflect the solution vs. immobilized states of ChoKα, with the latter more representative of the membrane-bound state where these proteins are likely to interact in cells. These results confirm the importance of the proline residues at position 61 and 62 to facilitate binding between ChoKα and the SH3 domain of Src.

### Crystal structure reveals how the polyP region of ChoKα interacts with c-Src-SH3

In order to obtain molecular insight into the binding mode between the ChoKα poly-proline region and the c-Src SH3 domain, we initiated crystallographic studies. We first attempted to co-crystallize the c-Src SH3 domain in the presence of a peptide that corresponds to the region that was identified by the SDS-PAGE/SPR experiments as being central to this interaction, residues 58–67. Unfortunately, we did not obtain crystals using this strategy. Therefore, we pursued an alternative approach where the His-SUMO-c-Src-SH3 construct was fused on it its C-terminus to peptides representing the poly-proline region of ChoKα responsible for the interaction. Three expression constructs were assembled, which encompassed fusion peptides derived from ChoKα residues 57–67, 56–69, and 60–69. Each fusion protein was expressed and purified, and the His-SUMO tag was cleaved to yield pure protein. Of the three proteins, only the Src-SH3(84–137)-ChoKα(60–69) fusion protein formed crystals and was subsequently used for structure determination.

**Figure 6:**
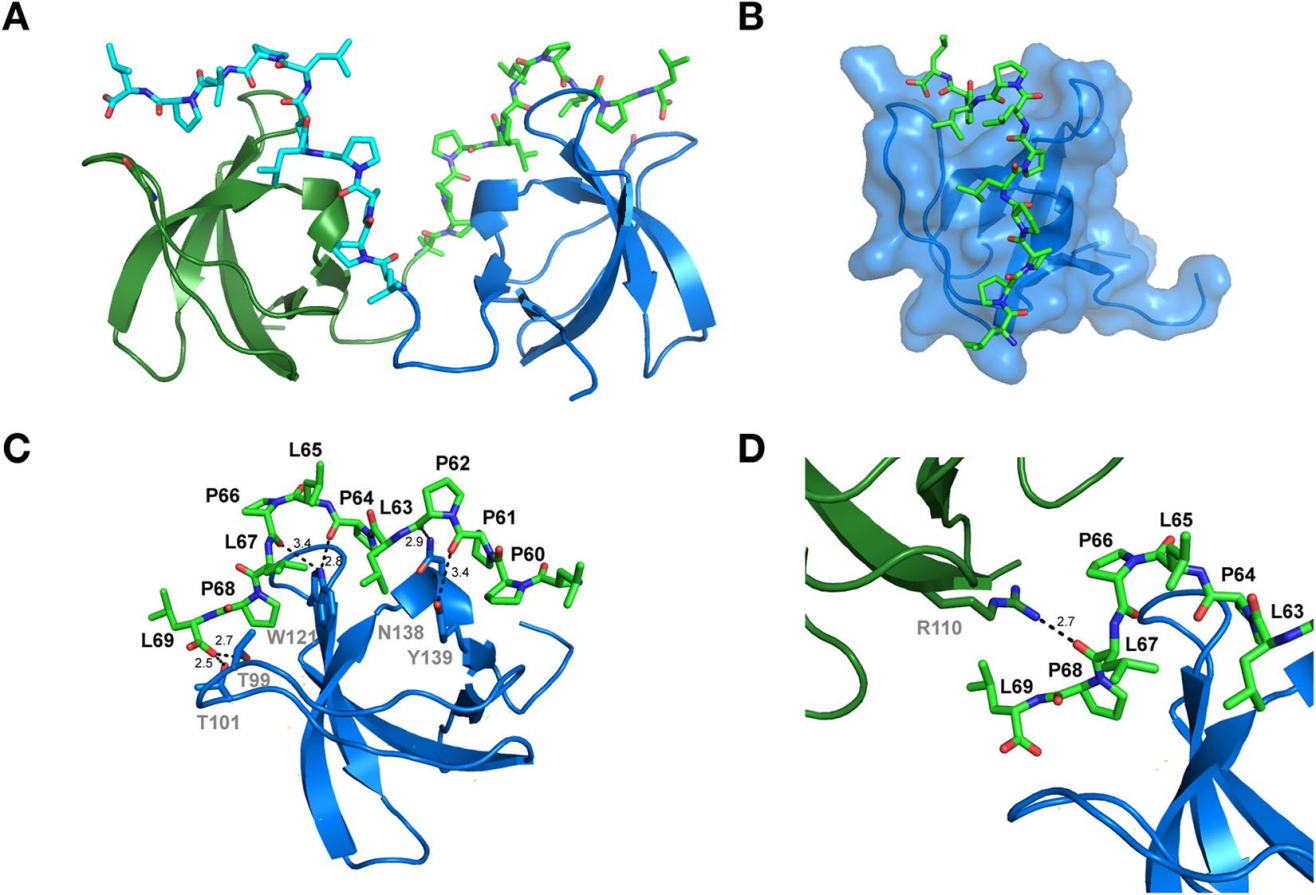
Crystal structure of c-Src-SH3 fused to the ChoKα (60–69) proline-rich peptide. (**A**) The crystal structure reveals a non-biological assembly unit with the peptide of domain A binding to domain B and vice-versa. (**B**) Residues 60–69 of ChoKα bind across the SH3 domain surface as a polyproline type II helix as observed in previous SH3:peptide structures (Ref 24). (**C**) Key interactions between ChoKα (60–69) and c-Src-SH3 show why prolines 61 and 62 are crucial for the interaction, as described in Fig. 2. (**D**) Crystal contacts strengthen the interaction of the peptide and the SH3 domain. Though ChoKα (60–69) does not contain an arginine, the interaction is stabilized by Arg110 from protomer A of an adjacent unit cell.

Crystals of the SH3-ChoKα(60–69) fusion protein diffracted to 1.5 Å resolution; data collection and refinement statistics can be found in Table 1. Molecular replacement was performed to solve the structure using the *Gallus gallus* c-Src SH3 domain in complex with a poly-proline peptide derived from Hepatitis C non-structural protein 5 A (NS5A) as a model. A model of the resulting structure is presented in Fig. 6. The CC_1/2_ parameter was used to select the high-resolution cutoff for this structure.

**Table 1:** Data collection and refinement statistics for c-SrcSH3_CK (60–69).

Structure PDB ID	c-SrcSH3_CK (60–69) 6C4S
**Data Collection**	
Space Group	*P2*_*1*_
***Cell dimensions***	
a, b, c (Å)	31.10 38.80 57.90
β (°)	95.7
Resolution (Å)	30.0–1.50 (1.59–1.50)*
R_merge_ (%)	8.4 (70.8)
I/σI	10.04 (2.84)
CC_1/2_ Completeness (%)	99.3 (76.7) 97.6 (95.9)
Reflections Unique Reflections	67389 21743
**Refinement**	
Resolution (Å)	1.50
R_w_/R_f_ (%)	18.7/22.6
***No***. ***Atoms***	
Protein	1158
Water Zn^2+^	138 2
***R***.***M***.***S***.***D***	
Bond Length (Å)	0.019
Bond Angles (°)	1.95
***Ramachandran Plot*** (***%***)	
Most Favored	97.9
Additionally Allowed	2.1
Disallowed	0

*Numbers in parenthesis are for the highest resolution shell.

Though SH3 domains function as monomers in solution, the SH3-ChoKα(60–69) fusion protein crystallized as a dimer. The ChoKα-derived peptide of protomer A bound to the c-Src SH3 domain of protomer B and vice versa (Fig. 6A). There were no substantial interactions between the protomers besides their interaction with the respective peptide region. Clear electron density was observed for the entire length of both peptides, although the side chains in the protomer B/peptide A protomer had better defined electron density. Subsequent structural analysis was done using this protomer/peptide.

The ChoKα (60–69) polyproline peptide associates with the hydrophobic surface of the c-Src-SH3 domain in a manner similar to other PxxP peptides with c-Src-SH3 (Fig. 6B). The ChoKα (60–69) peptide adopts a PPII helical conformation that binds in an orientation echoing a Class II^33^ SH3-binding peptide^34^ in terms of orientation (i.e. C←N), despite the lack of an arginine residue in the peptide region. Notably, peptide residues corresponding to ChoKα Pro61 and Pro62 make direct contacts with the c-Src-SH3 domain, with their backbone carbonyl groups interacting with the side chains of Asn 138 and Tyr139 (Fig. 6C). Another important interaction occurs between Trp121 and the backbone carbonyls of both Pro64 and Pro66. Mutation of SH3 Trp121 resulted in a substantial loss of ChoKα binding to the Src SH3-SH2-linker protein, highlighting the importance of this interaction (data not shown). Finally, added stability for the peptide is likely a result of the C-terminal carboxy group, which interacts with both Thr99 and Thr101 on SH3.

As previously noted, ChoKα (60–69) does not contain a lysine or arginine residue, which often determines the binding orientation of peptide ligands to SH3 domains. Our crystal structure shows that Arg103 of protomer A from an adjacent unit cell helps stabilize the protomer B/peptide A interaction by forming a salt bridge with the carbonyl backbone of Leu67 (Fig. 6D). There is no equivalent crystal contact for protomer A/peptide B, which may explain why the electron density for peptide B is not as pronounced, though still clearly discernable. We speculate that basic residues present in nearby domains of c-Src or additional binding partners may lend further stability to the complex and enhance binding.

### Comparison of ChoKα (60–69) vs c-Src-SH3 bound to VSL12, a well-known optimized ligand

**Figure 7:**
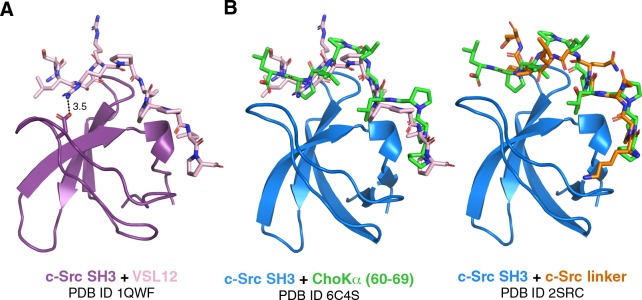
Comparison of c-Src SH3 in complex with optimised peptide VSL12 and ChoKα (60–69) or the linker region of c-Src. (**A**) The structure of c-Src SH3 in complex with the optimised peptide VSL12, as determined from solution NMR (PDB ID 1QWF). Note the interaction between Asp99 of the SH3 domain and Arg6’ of the peptide. (**B**) The c-Src SH3 domain in complex with ChoKα (60–69), reported here, vs. the VSL12 peptide (pink, left) or the c-Src linker (orange, right, from PDB ID 2SRC).

The autoregulation of c-Src is partially mediated by the intramolecular interaction of its SH3 domain with the SH2-kinase linker region, which adopts a PPII-like conformation in the context of the inactive kinase^35^. Previous studies have developed a peptide designated VSL12, which has high affinity for SH3 domains of SFKs^32^. Moroco *et al*. showed that VSL12, a poly-proline peptide with the sequence VSLARRPLPPLP, has high micromolar affinity for c-Src SH3. Furthermore, Arg6’ of the peptide interacts with Asp99 of c-Src SH3 (PDB ID: 1QWF, Fig. 7A). In the same vein, we speculate that the poly-proline region of ChoKα, possibly in concert with the SH2 domain or another Arg-containing regulatory element, may modulate internal SH3:linker interaction of c-Src.

We also compared our structure to a previously published c-Src SH3 domain in complex with the VSL12 peptide (PDB ID: 1QWF), as well as the “closed” form of full-length c-Src with a focus on the interaction between the linker region and the SH3 domain (PDB ID: 2SRC). Interestingly, the ChoKα-derived peptide binds with the opposite directionality of VSL12 (i.e. class I vs class II^34^) but the same as the linker region (class I) (Fig. 7B). As suggested previously, it is important to consider the full context of binding, as additional domains or binding partners may further modulate the c-Src + ChoKα interactions (see Fig. 6D). However, these comparisons suggest additional ways to modulate c-Src activity.

## Discussion

Several studies have demonstrated that inhibitors of ChoKα have a strong effect on *in vivo* models not only in cancer^15^ but also against malaria^6^, rheumatoid arthritis^36^ and more recently in inflammation^37^. The premise behind the inhibitor development program is that it is the enzymatic activity of ChoKα that is responsible for cancer growth and malignancy. However, the discrepancy between *in vitro* inhibition and actual effect on cancer cells suggests there is more to consider than just enzymatic activity. Ablation of ChoKα has a greater effect *in vivo* than simple inhibition^17^. A few studies have already suggested a role of ChoKα beyond that of a metabolic enzyme^4,5^. Its implication as an interactor in an EGFR/c-Src/ChoKα complex led to this biophysical investigation of how ChoKα and c-Src may interact^5^.

With this in mind, along with the well-established fact that SH3 domains are known to interact with PxxP motifs^31^, and because the disordered sequence between residues 50–75 of ChoKα revealed a proline-rich region, it seemed prudent to investigate whether the SH3 domain of c-Src interacted with ChoKα. We conducted *in vitro* biophysical studies of various ChoKα constructs in order to determine where and how this interaction was mediated.

Our work reveals that there is an interaction between the SH3 domain of c-Src and ChoKα. However, the activity of ChoKα had no bearing on the interaction with the SH3 domain; SH3 binding did not alter ChoKα activity, nor did lack of ChoKα activity affect c-Src SH3 binding. Additionally, SPR suggests this interaction is somewhat specific to the SH3 domain of c-Src, and does not interact with the SH3 domains of related Src-family kinase SH3 proteins. The K_D_ values obtained by SPR for the interaction of ChoKα with the isolated SH3 domain of Src were in the low to mid-micromolar range. However, it is important to consider that *in vivo*, weaker biological interactions may be favorable, as they may be desirable for modulation and disruption in the dynamic systems of the cell^38^.

Our crystallographic study shows that an arginine is not necessary to mediate the binding of the PxxP motif within ChoKα to c-Src-SH3, contrary to published studies^39^. However, an arginine or lysine residue coming from an adjacent domain (e.g. the SH2 domain or another protein such as EGFR) may contribute to the stability of the peptide-domain interaction. This prediction is based on the observation that in the crystals, for one of the protomers in the asymmetric unit, an arginine residue from an adjacent SH3 domain stabilizes the ChoKα peptide. In other words, the arginine in this case is supplied in *trans*, and not in *cis* as in other SH3 domains crystallized with PxxP peptides, such as the VSL12 peptide^32^. Though a target peptide without an Arg residue is not unheard of, it is rare, as the basic residue enhances the stability of the peptide-domain interaction and helps mediate specificity^39^. It is possible that the reason this interaction has not been previously isolated from phage display is that the non-arginine containing peptides do not have a high enough affinity to pass the selection threshold^40^.

Our findings have fresh implications for the use of ChoKα drug discovery. As previously described, inhibition vs. ablation of ChoKα in tumor cells and xenograft models can lead to different physiological outcomes^4^. Most published inhibitors bind in the choline-binding pocket of ChoKα, which allows for specificity of the inhibitor and blocks the enzymatic reaction^12,41^. Our work, combined with previous *in vivo* reports, suggest an interaction between EGFR, c-Src, and ChoKα, which is likely to have additional downstream effects beyond ChoKα’s traditional role in the Kennedy pathway^5^. Understanding this interaction, its specificity, downstream effects, and physiological relevance could be an additional piece of the puzzle in the search for ideal drug development candidates for ChoKα.

One such example may be found in previous work from our group on the ChoKα inhibitor, TCD-717^42,43^. This compound was shown to ablate both ATPase and kinase activity of the enzyme. However, crystallographic studies of ChoKα in complex with TCD-717 gave clear evidence that the inhibitor did not bind in the choline-binding pocket, as is the case for previously published inhibitors. Instead, it bridges across the N- and C- terminal lobes of the catalytic region of the enzyme (i.e. residues 80–457). We speculated that because TCD-717 binds in this manner, it has the added benefit of disrupting the protein surface, blocking or reducing the ability of ChoKα to perform its additional protein binding function.

The β isoform of ChoK is structurally similar to ChoKα, but lacks the poly-proline region found in the α isoform^12^. ChoKβ has been largely implicated in Rostrocaudal Muscular Dystrophy, which is a result of a total deletion of ChoKβ^44^, and more recently as a modulator of bone homeostasis^45^. Interestingly, while depletion of ChoKα leads to cell death^46^, the deletion of ChoKβ does not appear to result in the same phenomenon. Additionally, is had been shown in mice that deletion of the *chka* gene that encodes for ChoKα is embryonic lethal, while *chkb* deletion for ChoKβ is not^47^. It is possible the additional protein-protein interaction role of ChoKα is what leads to more dramatic cellular outcomes upon deletion compared to its β counterpart.

The elucidation of the specific interaction between c-Src and ChoKα is just the beginning of characterizing this interaction and defining its importance *in vivo*. Further studies will address whether the disruption of c-Src and ChoKα can reduce the malignant profile of cancer cell lines. Additionally, it is worth considering to what extent this protein binding function affects cell proliferation and motility compared to ChoKα’s role as a metabolic enzyme. Finally, it would be prudent to investigate the molecular mechanism by which EGFR interacts with these proteins, creating a better understanding of how the three-way protein interaction affects cells. Experiments examining co-localization of the proteins in this complex could give insight to how ChoKα fits into the deregulation of cellular behavior in a broader sense.

In summary, ChoKα is a prime target for chemotherapeutic drug development due to its well-known role in many forms of cancer. Therefore, a better understanding of its larger role in the cell is crucial for designing drugs that target *all* of its effects in cancer progression. New structural data presented here reveal that the SH3 domain can bind the N-terminal region of ChoKα. Based on this finding, we hypothesis that ChoKα may impact cellular function in a manner that is independent of its enzymatic activity. In addition, our data strengthen the link between ChoKα and c-Src, which has also been implicated in tumor growth and metastasis. Better understanding of the alternative roles that ChoKα and other metabolic enzymes will undoubtedly be crucial for discerning cellular responses to future generations of drugs, and offer new targets for drug development libraries.

## Materials and Methods

### Materials

All chemicals were reagent or molecular biology grade. Platinum *Pfu* polymerase and DNA size markers were from ThermoScientific. dNTPs were from Promega. Restriction endonucleases and Sticky-End Master Mix Ligase were from New England Biolabs. Agarose gel purification, PCR product clean-up, and plasmid mini prep columns were all products of Qiagen. Human liver cDNA library was a product of ResGen (Invitrogen). All oligonucleotides were supplied by IBA GmbH, Göttingen, Germany. Choline and pyruvate kinase were from Sigma. ATP, NADH, phosphoenolpyruvate, and lactate dehydrogenase were purchased from Roche. SDS-PAGE gels were purchased from GenScript, and run using the supplied MOPS buffer. CM5 sensor chip for SPR was from GE Healthcare Life Sciences and used on a Biacore T200 system.

### Gene synthesis, cloning, protein expression, and purification of ChoKα constructs for kinetic, biophysical, and structural analysis

The Choline Kinase α (UniProt P35790) constructs were derived from the previously made GST-tagged Choline Kinase α1 FL or ∆49 vectors available in the lab^3^. For these constructs, the NdeI/BamHI sites at the 5’ and 3’ end of the insert were digested (restrictions enzymes *Nde*I and *Bam*HI-HF from New England BioLabs) and re-ligated into a modified pET14b vector containing a His_6_-tag followed by a SUMO protease site. To create the ∆79 construct, the FL WT construct in the modified pET14b vector was used as a template for a DNA amplification primer containing an *Nde*I site in the forward direction (*ChoK_del79_NdeI_F*), while T7 Terminator standard primer was used for the reverse direction (See Table S1 for primers).

For D306A kinase-inactive mutants (*ChoK_D306A_F/R*) and P—>A protein binding mutants (*ChoK_P5960A_F/R; ChoK_P6*1*62A_F/R; ChoK_P7273A_F/R*), a QuikChange Site-Directed Mutagenesis kit was used according to the kit protocol (Table S1).

The GST-tagged cSrc-SH3 (87–145) domain construct was a generous gift of the Kay Lab^48^. Similarly to the ChoKα constructs, the gene insert was amplified with forward and reverse primers (*F_NdeI_cSrcSH3; cSrc_S137term_R*) to create an NdeI/BamHI-flanked insert for ligation into the His_6_-SUMO modified pET14b vector. The result was a vector containing His_6_-SUMO-cSrc-SH3(87–137).

For crystallographic purposes, peptides were added to the end of this construct by amplifying in the forward direction with T7 Short primer and with reverse primers containing the final peptide and a BamHI site to be re-ligated into the modified pET14b vector^49^. Several constructs were developed for screening (*cSrc_SH3_ChoK59–67_R; ALP_insert_F/R; PL_insert_F/R*), with the final version containing a sequence of His_6_-SUMO-cSrcSH3(87–137)- ChoKα (57–69).

The resulting choline kinase DNA constructs, after verification by Sanger sequencing, were transformed into the Rosetta (DE3)pLysS *E*. *coli* strain. Cells were grown at 37 °C in 2xYT medium, which was supplemented with 100 μg/mL Ampicillin (Amp) and 34 μg/mL Chloramphenicol (Chlor). Protein expression was induced with 0.1–0.3 mM isopropyl β-D-1-thiogalactopyranoside (IPTG) once an OD_600nm_ of 1.0 was reached and cultured overnight at 22 °C. The cSrc-SH3 constructs were verified by Sanger sequencing, transformed into Rosetta (DE3)pLysS E.Coli cells, and grown in autoinducible media at 25° for 18 hours^50^.

All cells were harvested by centrifugation, washed with 200 mM KCl, 25 mM Tris pH 7.5, and 10 mM MgCl_2_, and centrifuged at 5000 rpm for 20 min before storage at −20 °C. After thawing, cells were lysed by sonication in 25 mM Tris pH 7.5, 200 mM KCl, 10 mM MgCl_2_, 10 mM imidazole pH 7.5, 10% glycerol, 1% Triton X-100, and 1 mM PMSF. Lysed cells were centrifuged at 20,000 rpm for 30 min. Clarified supernatant was loaded onto 5 mL HisTrap HP Ni^2+^ Sepharose column (GE Healthcare)^51^, and the column washed with 25 mM Tris pH 7.5, 500 mM NaCl, supplemented with 25 mM and then 50 mM imidazole. Protein was eluted in a single fraction in the same buffer supplemented with 500 mM imidazole.

If necessary, the His_6_-SUMO tag was cleaved with SUMO protease while dialyzing against 25 mM Tris, pH 7.5, 500 mM NaCl, 10 mM imidazole, and the tag was removed by loading the sample back onto a nickel column The collected flow-through fraction containing cleaved, purified protein was concentrated to 5 mL. Uncleaved protein for the pull-down experiments went immediately to concentration to 5 mL.

Once concentrated, protein was injected onto S-200 gel filtration column (GE Healthcare)^51^ equilibrated with 25 mM Tris-HCl pH 7.5, 500 mM NaCl, 3 mM DTT, and 1 mM EDTA. To confirm the purity, collected fractions were analyzed by SDS-PAGE and detected with Coomassie Brilliant Blue staining. All fractions containing purified protein were pooled, concentrated to 1–1.5 mL final protein, and stored at −80 °C.

### SDS-PAGE pull-downs of choline kinase constructs and His-SUMO-cSrc (87–137)

Concentration of purified protein from the size exclusion column was measured using nanodrop using the extinction coefficients as calculated from ExPASy. The His-SUMO tagged cSrc-SH3 was used as bait, while size-exclusion purified cleaved ChoKα constructs were used as probes. Equimolar amounts of each protein were incubated together at a total volume of 200 µl in a 1.7 mL eppendorf tube for 30 minutes in a buffer of 200 mM KCl, 30 mM imidazole pH 8.5, 25 mM Tris pH 8.5, and 10 mM MgCl_2_. Here, a sample was taken for the SDS-PAGE gel to show both proteins were present. While incubating the proteins, 500 µl of Ni^2+^ beads were prepared by washing 3x in 1 mL of wash buffer at 5 min for 1600 rpm in a microcentrifuge and decanting off the liquid each time, then preparing a final bead slurry of 50:50 beads:buffer. 50 µl of this slurry was added to the incubated tubes and allowed to further incubate for 30 min with rocking^52^.

Each tube was spun for 10 min at 1600 rpm and the liquid decanted. They were then washed 3x with 200 µl buffer for 5 min at 1600 rpm. Final bead-protein samples were resuspended with 25 µl of buffer, which was mixed into a slurry and loaded on the SDS-PAGE gels. Gels were run for 33 min at 200 V, then visualized with Coomassie brilliant blue.

Quantification of gel intensities was done using ImageJ software^53^. In brief, the gel bands were selected and analyzed for their area intensity using the plot lanes tool in ImageJ. These values were compared as a ratio of area_ChoK_:area_His-tagged_ to determine the relative amount of ChoKα pulled down by the bead-bound His-tagged construct of interest. The ratios were then compared to determine reduction in binding.

### SPR

Recombinant, purified Src-family kinase SH3 domain proteins were immobilized on a single carboxymethyl dextran CM5 biosensor chip (Biacore) with phosphate-buffered saline, pH 7.5, as running buffer at a flow rate of 30 µL/min. The remaining channel was used as the reference control. Recombinant ChoKα proteins were then injected in triplicate over a range of concentrations (0.1 to 30 μM) for two min followed by a 2 min dissociation phase in running buffer only. Stead-state K_D_ values were estimated by non-linear curve fitting of plots of RUmax at each analyte concentration using GraphPad Prism. Alternatively, recombinant ChoKα proteins were immobilized on the chip surface, and recombinant purified c-Src regulatory domain proteins were injected as analytes. Kinetic rate constants were calculated from reference-corrected sensorgrams using the BiaEvaluation software package, and were best fit by a two-state induced fit binding model. Equilibrium dissociation constants (K_D_) were then determined from the resulting kinetic rate constants. All SPR data were collected using a Biacore T-100 4-channel SPR instrument (GE Life Sciences).

### Kinetic assay of choline kinase α

Choline kinase activity was assayed spectrophotometrically using a modified pyruvate kinase/lactate dehydrogenase-coupled system^54^. The reaction buffer contained 100 mM Tris-HCl pH 7.5, 100 mM KCl, 10 mM MgCl_2_, 0.5 mM phosphoenolpyruvate, 0.25 mM NADH, four units of pyruvate kinase, and seven units of lactate dehydrogenase; final reaction mixture also contained purified enzyme, and substrates to a total volume of 1 ml. The reaction performed at 37 °C was initiated by the addition first of enzyme, to monitor the latent ATPase rate of the enzyme. This was followed by addition of choline to monitor the kinase reaction rate. ADP formation was followed spectrophotometrically using a Varian Cary 50 spectrophotometer by measuring the decrease of NADH at 340 nm. An IC_50_ curve was determined by analyzing the data in GraphPad Prism 6.0.

### Crystallization of the c-Src SH3 domain fused to ChoK (60–69): c-SrcSH3-ChoK (60–69)

All *c-SrcSH3-ChoK* (*60–69*) crystals were grown at 20 °C using sitting drop vapor diffusion method^55^. Crystals were obtained from the JCSG+ screen from Qiagen, in condition #55: 0.2 M Zinc Acetate, 0.1 M Imidazole pH 8.0, and 20% w/v PEG 3000. Drops of 2 µl protein at 10 mg/ml were set at a 1:1 ratio with reservoir solution. Crystals took 2–3 weeks to appear as branched rectangular pyramids. A facet of one of the pyramids was broken off for data collection during mounting in order to have a single crystal. Prior to data collection, the crystals were cryoprotected in mother liquor containing 30% ethylene glycol.

### Data collection and structure determination of c-SrcSH3-ChoK (60–69)

Diffraction data for *c-SrcSH3-ChoK* (*60–69*) was obtained at the Life Sciences Collaborative Access Team ID beamline 21-ID-F at the Advanced Photon Source, Argonne National Laboratory (wavelength, 0.979 Å; temperature, 100 K) (refer to Table 1 for data collection and refinement statistics). Data processing was executed using XDS^56^. The *c-SrcSH3-ChoK* (*60–69*) structure was solved by MOLREP^57^ using the structure of c-Src-SH3 with the NS5A peptide, PDB ID: 4QT7^58^, as a model. The peptide region from choline kinase was initially built using ARP^59^. Further refinement of the model was done using REFMAC5^60^ with manual rebuilding performed in Coot^61^. All figures of structures were generated using MacPyMOL (PyMOL™ Molecular Graphics System, version1.7.2.3; Schrodinger, LLC).

## Supplementary information

Supplementary Data

## Data Availability

The crystal structure reported here has been deposited at the PDB under accession code 6C4S.
