# Supplementary material for: Molecular basis for the interaction between human choline kinase alpha and the SH3 domain of the c-Src tyrosine kinase
Source: Sci Rep. 2019 Nov 19;9:17121. doi: 10.1038/s41598-019-53447-0 (PMC6864063; doi:10.1038/s41598-019-53447-0)
Supplement: Supplementary file 1 — Supplementary Data [file 41598_2019_53447_MOESM1_ESM.docx]

**Supplementary Information**

Molecular basis for the interaction between human choline kinase alpha and the SH3 domain of the c-Src tyrosine kinase

**Stefanie L. Kall^1^, Kindra Whitlatch^2^, Thomas E. Smithgall^2^, and Arnon Lavie^1,3,^***

From the ^1^Department of Biochemistry and Molecular Genetics, University of Illinois at Chicago, Chicago, Illinois, 60607, USA.

^2^Department of Microbiology and Molecular Genetics, University of Pittsburgh School of Medicine, Pittsburgh, Pennsylvania 15219, USA.

^3^The Jesse Brown VA Medical Center, Chicago, Illinois 60612, USA.

Running title: *CHKA- Src interaction*

*Address correspondence to: Arnon Lavie, PhD, 900 South Ashland Avenue, MBRB room 1108, Chicago, IL, 60607. Phone: (312) 355-5029; Fax: (312) 355-4535; E-mail: Lavie@uic.edu

Table S1: List of primers for mutagenesis

| **Primer Name** | **Target Vector** | **Primer Sequence** |
| --- | --- | --- |
| ChoK_del79_NdeI_F | ChoK (FL) | ATA TGC CGC CGC AGC CGC ATA TGG ACG AGC AGC CGG AGC C |
| ChoK_D306A_F | ChoK (FL, ∆49, ∆79) | TCC AGT TGT ATT TTG TCA TAA TGC CTG TCA AGA AGG TAA TAT CTT GT |
| ChoK_D306A_R | ChoK (FL, ∆49, ∆79) | ACA AGA TCT TAC CTT CTT GAC AGG CAT TAT GAC AAA ATA CAA CTG GA |
| ChoK_P5960A_F | ChoK (FL or ∆49) | GCG CTG CCC GCT GCG CCG CCG C |
| ChoK_P5960A_R | ChoK (FL or ∆49) | GCG GCG GCG CAG CCG GCA GCG C |
| ChoK_P6162A_F | ChoK (FL or ∆49) | CCC CCT CCG GCG GCG CTG CCG C |
| ChoK_P6162A_R | ChoK (FL or ∆49) | GCG GCA GCG CCG CCG GAG GGG G |
| ChoK_P7273A_F | ChoK (FL or ∆49) | CTG CCC CAG GCC GCG CCG CCG C |
| ChoK_P7273A_R | ChoK (FL or ∆49) | GCG GCG GCG CGG CCT GGG GCA G |
| F_NdeI_cSrcSH3 | cSrc-SH3 (87-145) | GGG AAT TCC ATA TGA CCA CCT TTG TGG C |
| cSrc_S137term_R | cSrc-SH3 (87-145) | CTC AGC CTG GGA TCT AGT CGG AGG GCG C |
| cSrc_SH3_ChoK59-67_R | cSrc-SH3 (S137*) | CGC GGA TCC TTA AAG GGG AAG TGG TAA GGG TGG CGG GGG TCC TCC TCC GTC GGA GGG CGC CAC |
| ALP_insert_F | cSrc-SH3_CK(59-67) | TCC GAC GGA GGA GGA GCG CTG CCG CCC CCG CC |
| ALP_insert_R | cSrc-SH3_CK(59-67) | GGC GGG GGC GGC AGC GCT CCT CCT CCG TCG GA |
| PL_insert_F | cSrc-SH3_CK(59-67) | CTT ACC ACT TCC CCT TCC GCT GTA AGG ATC CGG CTG CT |
| PL_insert_R | cSrc-SH3_CK(59-67) | AGC AGC CGG ATC CTT ACA GCG GAA GGG GAA GTG GTA AG |


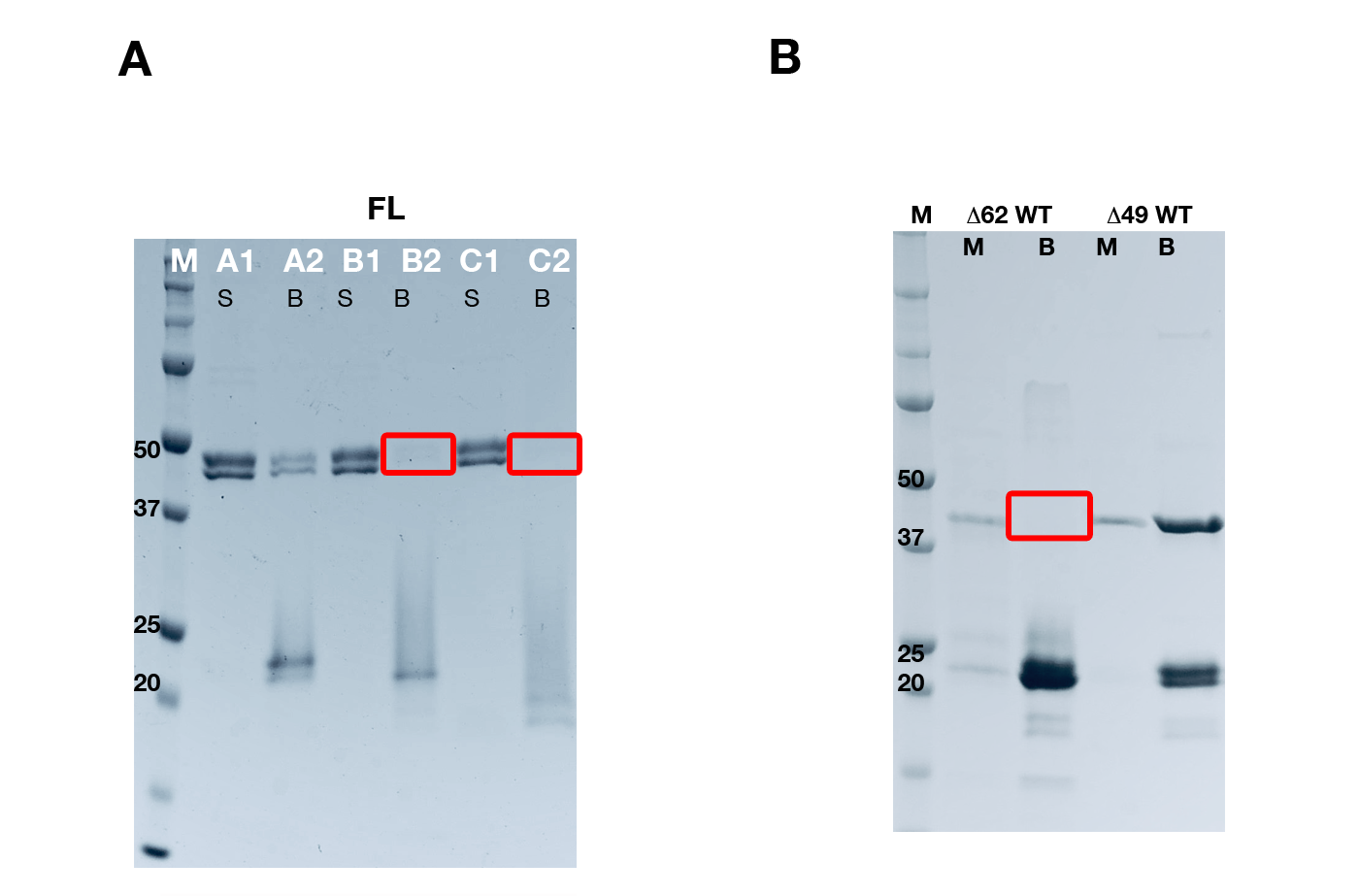


**Figure S1:** **A**- Pull-down controls for the SDS-PAGE experiments. Full length ChoKα (52.2 kDa) vs. His-SUMO constructs for comparison. Lanes- M: molecular weight ladder; A1: ChoKα + His_6_-SUMO-cSrcSH3(87-137) (19.6 kDa) supernatant; A2: ChoKα + His_6_-SUMO-cSrcSH3(87-137) Ni^2+^ beads; B1: ChoKα + His_6_-SUMO-MLK3SH3(41-105) (20.2 kDa) supernatant; B2: ChoKα + His_6_-SUMO- MLK3SH3(41-105) Ni^2+^ beads; C1: ChoKα + His_6_-SUMO (14.4 kDa) supernatant; C2: ChoKα + His_6_-SUMO- Ni^2+^ beads. Red boxes denote where ChoKα would be expected (S-supernatatant of mixture decanted before washing; B- bead pull-down) **B**- SDS-PAGE pull-down for additional construct ChoKα∆62 (MW: 46.2 kDa) and ChoKα∆49 vs. ChoKα + His_6_-SUMO-cSrcSH3(87-137) (M- mixture; B- bead pull-down).
